# The clinical applications of ensemble machine learning based on the Bagging strategy for in-hospital mortality of coronary artery bypass grafting surgery

**DOI:** 10.1016/j.heliyon.2024.e38435

**Published:** 2024-09-26

**Authors:** Kai Xu, Lingtong Shan, Yun Bai, Yu Shi, Mengwei Lv, Wei Li, Huangdong Dai, Xiaobin Zhang, Zhenhua Wang, Zhi Li, Mingliang Li, Xin Zhao, Yangyang Zhang

**Affiliations:** a Department of Cardiovascular Surgery, Qilu Hospital of Shandong University, Jinan, Shandong, PR China; b Institute of Thoracoscopy in Cardiac Surgery, Qilu Hospital of Shandong University, Jinan, Shandong, PR China; c Department of Thoracic Surgery, Sheyang County People's Hospital, Yancheng, Jiangsu, PR China; d College of Information Science, Shanghai Ocean University, Shanghai, PR China; e Department of Cardiovascular Surgery, East Hospital, Tongji University School of Medicine, Shanghai, PR China; f Department of Thoracic Surgery, Xuzhou Cancer Hospital, Xuzhou, PR China; g Department of Cardiovascular Surgery, Shanghai Chest Hospital, Shanghai Jiao Tong University School of Medicine, Shanghai, PR China; h Department of Cardiovascular Surgery, Jiangsu Province Hospital, The First Affiliated Hospital of Nanjing Medical University, Nanjing, Jiangsu, PR China; i Department of Cardiovascular Surgery, The General Hospital of Ningxia Medical University, Yinchuan, Ningxia, PR China

**Keywords:** Machine learning, In-hospital mortality, Coronary artery bypass grafting, Prediction

## Abstract

**Background:**

Machine learning (ML) has excelled after being introduced into the medical field. Ensemble ML models were able to integrate the advantages of several different ML models. This study compares the ensemble ML model's and EuroSCORE II's performance predicting in-hospital mortality in patients undergoing coronary artery bypass grafting surgery.

**Methods:**

The study included 4,764 patients from three heart centers between January 2007 and December 2021. Of these, 3812 patients were assigned to the modeling group, and 952 patients were assigned to the internal test group. Patients from other two heart center (1733 and 415 cases, respectively) constituted the external test group. The modeling set data are trained using each of the three ML strategies (XGBoost, CatBoost, and LightGBM), and the new model (XCL model) is constructed by integrating these three models through an ensemble ML strategy. Performance of different models in the three test groups comparative assessments were performed by calibration, discriminant, decision curve analysis, net reclassification index (NRI), integrated discriminant improvement (IDI), and Bland-Altman analysis.

**Results:**

In terms of discrimination, the XCL model performed the best with an impressive AUC value of 0.9145 in the internal validation group. The XCL model continued to perform best in both external test groups. The NRI and IDI suggested that the ML model showed positive improvements in all three test groups compared to EuroSCORE II.

**Conclusions:**

ML models, particularly the XCL model, outperformed EuroSCORE II in predicting in-hospital mortality for CABG patients, with better discrimination, calibration, and clinical utility.

## Teaser

This study developed a new machine learning based model for predicting in-hospital mortality in patients undergoing CABG and to compare it with European System for Cardiac Operative Risk Evaluation II (EuroSCORE II), which is already widely used in clinical practice for cardiac surgery. However, recent studies have found limited application for EuroSCORE II in the Chinese population. In this study, 4,764 patients underwent primary isolated CABG from three medical centers were enrolled. Our model showed significant advantages over EuroSCORE II in predicting postoperative in-hospital mortality in CABG patients. Furthermore, the present study selected two different areas of groups to verify the generalization and obtained good results. This novel analytical approach may provide reliable information on cardiac operation in the near future.

## 1 Introduction

Coronary artery disease (CAD) is a common cardiovascular disease that causes severe damage to human health [1,2]. Coronary artery bypass grafting (CABG) is an effective surgical procedure for treating CAD, which has increased dramatically over the last decade [3,4]. Surgeons are becoming aware of the importance of risk assessment before cardiac surgery. European System for Cardiac Operative Risk Evaluation II (EuroSCORE II) is a quantitative risk assessment model widely used in preoperative risk assessment for cardiac surgery [5]. However, recent studies have found limited application for EuroSCORE II in the Chinese population [[6], [7], [8], [9]]. There is a need to enhance preoperative risk assessment models for patients undergoing CABG, particularly by developing tools that are more accurate and applicable to the Chinese population.

In recent years, machine learning (ML) has been increasingly used in the medical field [10]. ML can effectively model the complex associations between clinical features and patient prognosis and has attracted attention in clinical prediction models [11,12]. As the intersection of statistics and computer science, ML has unique advantages in processing massive, high-dimensional medical data and conducting predictive research [13]. Recently, several studies have explored the use of machine learning models to predict outcomes in patients undergoing CABG. For instance, studies have utilized ML methods such as random forests, support vector machines, and artificial neural networks to predict mortality and postoperative complications in cardiac surgery [[14], [15], [16]]. However, many of these studies either lacked validation in external test groups or focused on populations outside of China, limiting their generalizability to broader clinical settings [17]. Additionally, few studies have directly compared the performance of ML models with well-established risk scores like EuroSCORE II in large, multi-center patient cohorts.

Our study builds upon this body of research by developing an ensemble ML model that combines the strengths of XGBoost, CatBoost, and LightGBM for predicting in-hospital mortality in CABG patients. This approach distinguishes our work from prior studies, as we not only create a more robust predictive tool but also validate its performance across both internal and external patient groups, while providing a direct comparison with EuroSCORE II. Moreover, this is one of the first studies to apply ensemble ML strategies in predicting mortality for a large cohort of Chinese CABG patients.

## 2 Methods

### 2.1 Patients

#### 2.1.1 Modeling population

**Fig. 1:**
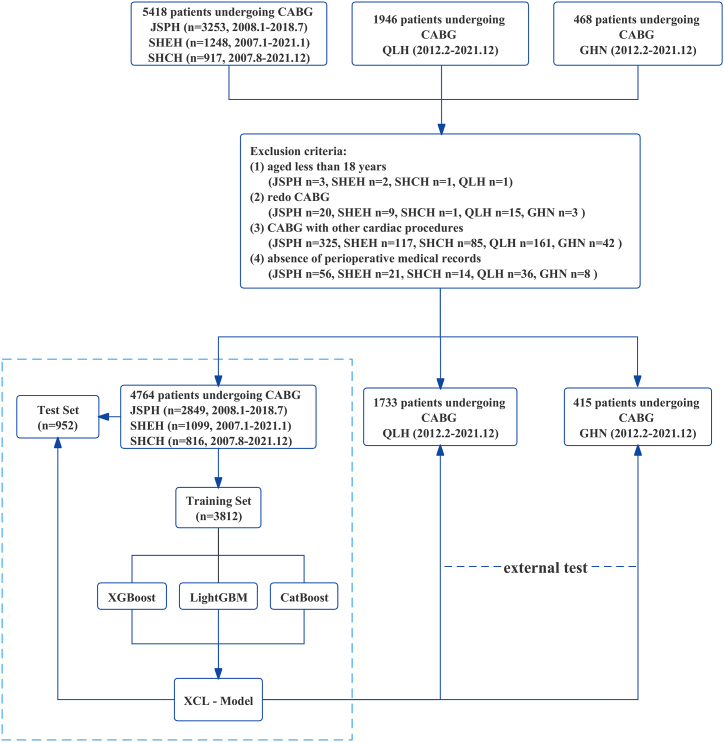
Flowchart of patient selection. (JSPH, Jiangsu Province Hospital, the First Affiliated Hospital of Nanjing Medical University; SHEH, Shanghai East Hospital, affiliated to Tongji University; SHCH, Shanghai Chest Hospital of Shanghai Jiao Tong University; QLH, Qilu Hospital of Shandong University; GHN, General Hospital of Ningxia Medical University; CABG, Coronary artery bypass grafting.)

From January 2007 to December 2021, 5,418 confidential patients undergoing CABG from three medical centers: Jiangsu Province Hospital (JSPH), Shanghai Chest Hospital (SHCH), and Shanghai East Hospital (SHEH). The inclusion criterion was: primary isolated CABG surgery for multiple coronary heart disease (CHD). The exclusion criteria were: (1) age less than 18 years; (2) redo CABG surgery; (3) combined with other cardiac procedures (e.g., valve, ventricular aneurysm, ventricular septum); (4) absence of perioperative medical records. 4,764 patients made up the study database. The study database was randomly assigned into modeling and test groups according to a 4:1 ratio. Ultimately, 3,812 patients formed the modeling group, and 952 patients formed the test group. The patients screening process was shown in Fig. 1.

**Fig. 2:**
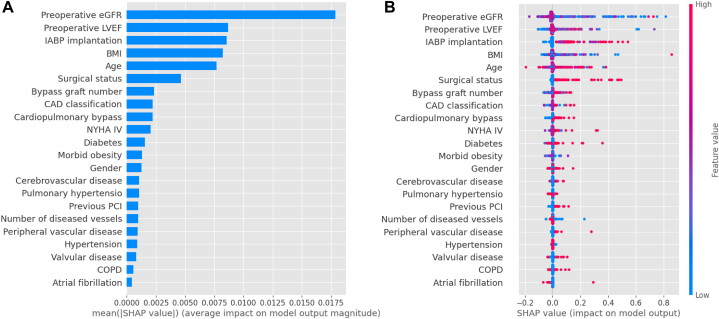
(A) The weights of variables importance and (B) the SHapley Additive exPlanation (SHAP) values of variables. (GFR, glomerular filtration rate; IABP, intra-aortic balloon pump; Scr, serum creatinine; LVEF, left ventricular ejection fraction; BMI, body mass index; NYHA, New York Heart Association; BSA, body surface area; CAD, coronary heart disease; COPD, chronic obstructive pulmonary disease; PCI, percutaneous coronary intervention.)

**Fig. 3:**
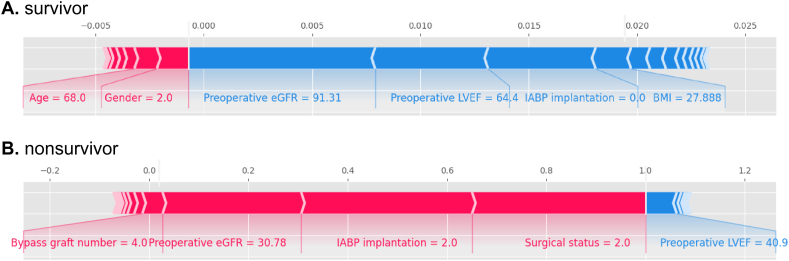
The individual SHAP force plots for patients who did (A) not survive and (B) survived.

#### 2.1.2 Validation population

Data from CABG patients in two other medical centers (Qilu Hospital of Shandong University (QLH) and General Hospital of Ningxia Medical University (GHN)), which are more than 500 km away from the previous three hospitals, were retrospectively collected according to the same inclusion and exclusion criteria from February 2012 to December 2021. Finally, the external test group consisted of 1,733 patients from QLH and 415 patients from GHN.

### 2.2 Informed consent

All included patients, or their legal representatives signed a written informed consent for all surgical procedures. In this retrospective study, there was no risk of disclosure of patient privacy or violating patient life and health. With the approval and consent of the Ethics Committees, all enrolled patients were exempted from informed consent for their participation. This study was approved by the Ethics Committees of hospitals (2017018 from SHEH; IS22063 from SHCH; KYLL20220584 from GHN; KYLL-202208-030 from QLH; 2022SR464 from JSPH) and was registered with the number of ChiCTR2200065598 (http://www.chictr.org.cn/).

### 2.3 Outcomes

The primary outcome event was in-hospital mortality, defined as all-cause death within 30 days after an operation or during postoperative hospitalization.

### 2.4 Machine learning design

#### 2.4.1 Data preprocessing

In this study, 4,764 case data from three medical centers (JSPH, SHEH, and SHCH) were used for modeling, of which 80 % were used as the training set for modeling, and 20 % were used as the validation set to verify the effectiveness of the XGBoost, CatBoost, LightGBM and XCL model. The XCL model, an ensemble model that combines XGBoost, CatBoost, and LightGBM, was specifically designed to integrate the strengths of each of these algorithms. Additionally, external data comprising 1,733 cases from QLH and 415 cases from GHN were used to assess the generalization ability of these models.

#### 2.4.2 Base learners

##### 2.4.2.1 XGBoost

XGBoost is an extension of the Gradient Boosting Decision Tree (GBDT); GBDT trains the newly added weak classifiers based on the negative gradient information of the loss function of the current model and then combines the trained weak classifiers into the existing model in an accumulative form. XGBoost has the characteristics of high training efficiency and good prediction effect [18]. In XGBoost, Shrinkage and Column Block are used to prevent overfitting, Cache-aware Access is used to improve computing speed, and Blocks for Out-of-core Computation are used to make more efficient use of system resources [19,20]. However, when it is directly applied to the risk assessment of cardiac surgeons, there are too many parameters, and the parameter adjustment is complicated and time-consuming.

##### 2.4.2.2 CatBoost

CatBoost, based on GBDT, can handle category features well. In CatBoost, the target statistic is used to solve the problem of feature volume explosion and category imbalance, the feature combination is used to effectively guarantee the cross-validation of numerical features, and Ordered Boosting is used to reduce the alleviation prediction bias effectively [21]. The CatBoost algorithm saves time for parameter adjustment, but the setting of different random numbers impacts the model prediction results.

##### 2.4.2.3 LightGBM

LightGBM (Light Gradient Boosting Machine), based on a histogram optimization algorithm, has the advantages of a smaller memory footprint and lower computational cost. In LightGBM, the Leaf-wise algorithm is used for multi-threaded optimization models as well as to control the complexity of the model, the one-sided gradient sampling algorithm is used to achieve a balance between reducing data and ensuring accuracy, and Exclusive Feature Bundling is used to reduce feature dimension while avoiding information loss [22]. However, it is sensitive to noise information and is ineffective in directly predicting mortality after cardiac surgery.

#### 2.4.3 Ensemble machine learning strategy

Ensemble learning combines multiple base learners into one strong learner to reduce prediction variance or bias. Usually, a series of “individual learners” are constructed. Then the corresponding strategies combine the individual learners to achieve the purpose of learning widely from others’ strong points [19].

Bagging is a commonly used ensemble learning method, which can train base learners in parallel independently of each other and then integrate the base learners into the strong learner. The Bagging strategy may reduce the variance of the model, thereby improving the model's stability. At the same time, the Bagging strategy also has strong generalization.

**Fig. 4:**
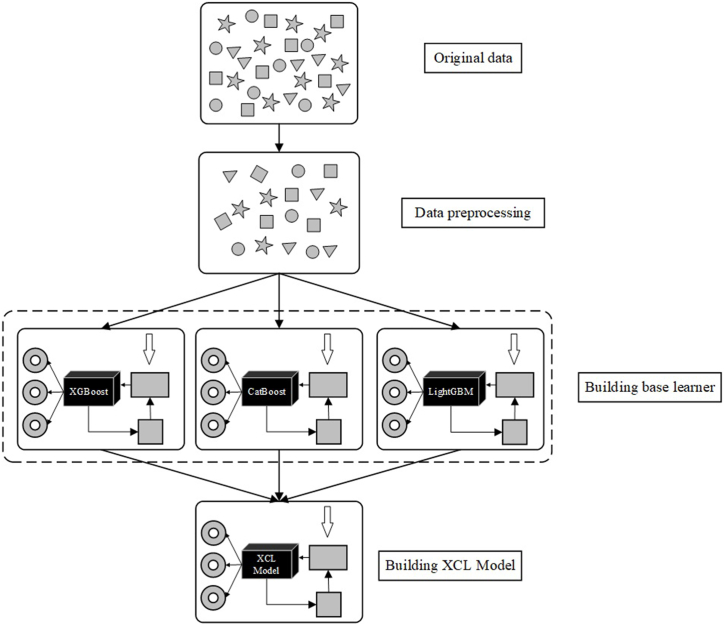
Flowchart of XCL model building.

In this study, the base learners were first built using XGBoost, CatBoost, and LightGBM. The three were combined using the Bagging strategy to build the XCL model. The process of making the XCL Model is shown in Fig. 4.

### 2.5 Statistical analysis

Measurement data with a normal distribution were presented as the mean ± standard deviation (S.D). Student's t-test was used for intergroup comparisons. Measurement data with a non-normal distribution were presented as the median and interquartile ranges (IQR). Mann–Whitney *U* test was used for intergroup comparisons. Count data were presented as relative numbers, and the Chi-square or Fisher exact test was used for intergroup comparisons.

Discrimination ability was measured by the receiver operating characteristic (ROC) curve [19]. [23]. The cut-off value is determined according to the maximum Youden index. The area under the ROC curve (AUC), sensitivity, and specificity was measured for each estimation method. *P* < 0.05 suggests good calibration. AUC >0.70, >0.75, and >0.80 indicate that discrimination is available, good, and excellent, respectively.

The performance of these risk evaluation models was assessed by comparing the expected and observed in-hospital mortality rates. Calibration (statistical precision) was analyzed by Hosmer–Lemeshow (H-L) goodness-of-fit statistic and Calibration plots. The H-L statistic measured the differences between expected and observed outcomes. A P-value greater than 0.05 means there is no evidence that this risk evaluation model is poorly calibrated.

Bland-Altman plots were used to estimate the agreement of models in pairs [24]. The Bland-Altman plot uses bias to portray the consistency of the two models. If the difference between the two models lies within the consistency bounds, then it is considered to be in good agreement. The higher the agreement between the two models, the closer the solid line representing the mean of the differences is to the dashed line with a zero value.

Decision curve analysis (DCA) was widely used to measure clinical utility [25]. DCA was used to measure the net benefit of different models for predicting in-hospital mortality. The net benefit for a given probability was calculated for each model as follows:

$$Net\;benefit=\frac{TP}{N}-\frac{FP}{N}\;\left(\frac{p}{1-p}\right)$$

where TP means true positive, FP means False Positive, N is the total number of subjects in the trial, and p is the threshold probability.

The net reclassification index (NRI) [26] and integrated discrimination improvement (IDI) [27] can further assess the predictive power of the two models concerning in-hospital mortality. When the value is greater than 0, it is an improvement, indicating that the new model has better predictive power for in-hospital mortality than the old one. When the value is less than 0, it is a negative improvement, indicating a decrease in the predictive ability of the new model. When the value is equal to 0, there is no difference in the predictive ability of the two models.

EpiData version 3.1 (EpiData Association, Odense, Denmark) statistical software was used to build a database, and Statistical Package for the Social Sciences (SPSS) version 25.0 (IBM Corp., Armonk, N.Y., USA) was used to sort up and analyze the data. Figures were plotted on GraphPad Prism 9.0 (GraphPad Software, CA) and R software 3.4.0. α = 0.05 was used as the threshold for significance for all statistical tests. The current study design follows the transparent report of a machine learning architecture and the Strengthening the Reporting of Observational Studies in Epidemiology (STROBE) reporting guideline.

## 3 Results

### 3.1 Baseline clinical characteristics

3,812 patients were eventually included in the modeling group. In the modeling group, the median age was 66 years old. There were 2,905 (76.20 %) males, 2,627 (68.91 %) patients with hypertension, 1,124 (29.51 %) patients with diabetes, 101 (2.64 %) patients receiving emergency operation, 379 (9.93 %) patients with valvular disease, and 409 (10.73 %) patients with a history of PCI. 74 (1.93 %) patients died within 30 days after the operation.

952 patients were included in the internal test group. Due to the random assignment, the differences in the main clinical characteristics between the two groups were not statistically significant (Table 1).

**Table 1:** Baseline clinical characteristics of modeling group and testing groups.

	Modeling Group (n = 3812)	Internal Test Group (n = 952)	*P* value	External Test Group			
				QLH (n = 1733)	*P* value	GHN(n = 415)	*P* value
Age(y)	66.00 (12.00)	66.00 (11.00)	0.053	66.00 (12.00)	＜0.001	62.86 (10.00)	＜0.001
Gender (male) (n, %)	2905 (76.20)	731 (76.79)	0.707	1242 (71.67)	＜0.001	284 (68.43)	0.001
Weight (kg)	68.00 (14.00)	69.00 (14.00)	0.061	70.00 (15.00)	＜0.001	69.01 (14.00)	＜0.001
Height (cm)	168.00 (12.00)	168.00 (12.00)	0.163	170.00 (12.00)	＜0.001	166.26 (12.00)	＜0.001
BMI (kg/m^2^)	24.65 (3.85)	24.91 (3.44)	0.611	24.65 (4.44)	0.091	24.95 (4.41)	0.050
BSA (m^2^)	1.86 (0.22)	1.86 (0.21)	0.886	1.90 (0.22)	＜0.001	1.86 (0.22)	0.063
Morbid obesity (n, %)	243 (6.37)	35 (3.68)	0.003	106 (6.12)	0.714	19 (4.58)	0.150
NYHA IV (n, %)	112 (2.94)	11 (1.16)	0.002	88 (5.08)	＜0.001	17 (4.10)	0.193
CAD classification			＜0.001		＜0.001		＜0.001
Stable angina (n, %)	959 (25.17)	290 (30.46)		117 (6.75)		36 (8.67)	
Unstable angina (n, %)	2382 (62.49)	500 (52.52)		1561 (90.08)		232 (55.90)	
AMI (n, %)	471 (12.34)	162 (17.02)		55 (3.17)		68 (16.39)	
Hypertension (n, %)	2627 (68.91)	677 (71.11)	0.188	1080 (62.32)	＜0.001	262 (63.13)	0.016
Diabetes (n, %)	1124 (29.51)	256 (26.89)	0.114	586 (33.81)	0.001	155 (37.35)	0.451
Cerebrovascular disease (n, %)	433 (11.36)	135 (14.18)	0.016	188 (10.85)	0.576	36 (8.67)	0.098
Preoperative Scr (μmol/l)	76.70 (26.00)	80.00 (26.00)	0.514	72.00 (20.00)	＜0.001	74.63 (24.30)	＜0.001
Preoperative eGFR (mL/min/1.73 m^2^)	77.32 (34.11)	75.26 (33.16)	0.047	84.63 (30.31)	＜0.001	90.11 (34.69)	＜0.001
Preoperative LVEF (%)	62.00 (8.88)	62.00 (8.88)	0.071	60.00 (13.00)	＜0.001	59.13 (16.99)	＜0.001
Number of diseased vessels (n, %)			0.565		＜0.001		0.229
1	170 (4.47)	37 (3.89)		16 (0.92)		9 (2.17)	
2	395 (10.35)	107 (11.24)		192 (11.08)		36 (8.67)	
3	3247 (85.18)	808 (84.87)		1525 (88.00)		309 (74.46)	
Peripheral vascular disease (n, %)	247 (6.49)	6 (0.63)	＜0.001	41 (2.37)	＜0.001	146 (35.18)	＜0.001
Surgical status (n, %)			0.769		＜0.001		＜0.001
Emergency	101 (2.64)	30 (3.15)		1 (0.06)		1 (0.24)	
Rescue	36 (0.94)	12 (1.26)		2 (0.12)		0 (0.00)	
Valvular disease (n, %)	379 (9.93)	44 (4.62)	＜0.001	52 (3.00)	＜0.001	27 (6.51)	0.030
IABP implantation (n, %)	105 (2.75)	11 (1.16)	0.004	81 (4.67)	＜0.001	21 (5.06)	0.009
COPD (n, %)	140 (3.67)	14 (1.47)	0.001	9 (0.52)	＜0.001	30 (7.23)	＜0.001
Atrial fibrillation (n, %)	127 (3.32)	21 (2.21)	0.073	33 (1.90)	0.003	13 (3.13)	0.765
Pulmonary hypertension (n, %)	564 (14.80)	5 (0.53)	＜0.001	563 (32.49)	＜0.001	5 (1.20)	＜0.001
Previous PCI (n, %)	409 (10.73)	83 (8.72)	0.068	95 (5.48)	0.501	42 (10.12)	0.703
Bypass graft number (n)	3.00 (1.00)	3.00 (1.00)		3.00 (1.00)		2.85 (0.00)	
Cardiopulmonary bypass (n, %)	1262 (33.12)	478 (50.21)	＜0.001	186 (10.73)	0.013	5 (1.20)	＜0.001
In-hospital mortality (n, %)	74 (1.93)	18 (1.89)	0.919	32 (1.85)	0.811	11 (2.65)	0.328

Abbreviation: QLH, Qilu Hospital of Shandong University; GHN, General Hospital of Ningxia Medical University; BMI, body mass index; BSA, body surface area; NYHA, New York heart association; CAD, coronary artery disease; AMI, acute myocardial infarction; Scr, Serum creatinine; eGFR, estimated glomerular filtration rate; LVEF, left ventricular ejection fraction; IABP, intra-aortic balloon pump; COPD, chronic obstructive pulmonary disease; PCI, percutaneous coronary intervention.

**Table 2:** Performance of each model for prediction.

	Cutoff	AUC (95%CI)	Sensitivity	Specificity	Accuracy
Internal test group					
XGBoost	0.0614	0.8078 (0.6925–0.9231)	0.7778	0.9058	0.9034
CatBoost	0.0934	0.8727 (0.7664–0.9791)	0.5556	0.9604	0.9531
LightGBM	0.1689	0.8986 (0.8130–0.9842)	0.8889	0.7880	0.7898
XCL	0.3011	0.9145 (0.8540–0.9729)	0.9444	0.7131	0.7173
EuroSCORE II	0.1335	0.7760 (0.6791–0.8730)	0.8889	0.5357	0.5426
External test group of QLH					
XGBoost	0.0214	0.7470 (0.6483–0.8458)	0.5938	0.8812	0.8754
CatBoost	0.1904	0.8375 (0.7451–0.9299)	0.7500	0.6818	0.6830
LightGBM	0.1127	0.7655 (0.6727–0.8582)	0.6250	0.8242	0.8217
XCL	0.2495	0.8585 (0.7763–0.9408)	0.8125	0.8260	0.8264
EuroSCORE II	0.1745	0.7534 (0.6537–0.8531)	0.6250	0.8236	0.8195
External test group of GHN					
XGBoost	0.0873	0.8804 (0.8248–0.9360)	0.8182	0.8490	0.8481
CatBoost	0.2020	0.7890 (0.6821–0.8960)	0.6364	0.8342	0.8219
LightGBM	0.1724	0.8155 (0.7080–0.9230)	0.7273	0.8342	0.8314
XCL	0.2721	0.8553 (0.7780–0.9326)	0.9091	0.6757	0.6814
EuroSCORE II	0.2420	0.7678 (0.6252–0.9103)	0.4364	0.8391	0.8283

Abbreviation: AUC, area under the curve; CI, confidence interval.

Two other cardiac centers, located far apart, joined the study to assess the external applicability of the models further. 1,733 from QLH and 415 patients from GHN formed the external test group.

### 3.2 Discrimination

**Fig. 5:**
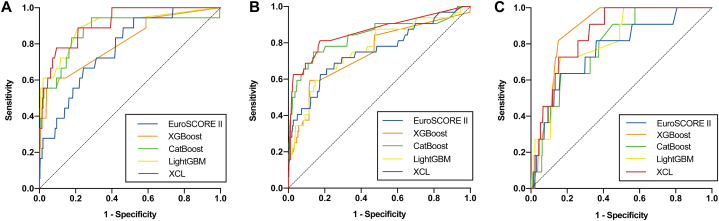
The receiver operating characteristic (ROC) curves of the risk evaluation models in (A) the internal test group and in external test groups of (B) QLH and (C) GHN.

In the internal test group, all four ML models (XGBoost, CatBoost, LightGBM, and XCL model) performed well in predicting in-hospital mortality, and all outperformed the EuroSCORE II. Among them, the XCL model performed the best, with an outstanding AUC value of 0.9145. In addition, XCL had the highest sensitivity (0.9444), while CatBoost had the best performance in terms of specificity and accuracy (specificity = 0.9604, accuracy = 0.9531). This phenomenon was also present in the two external test groups. In the QLH external test group, the EuroSCORE II had the general discriminatory power with an AUC of 0.7534, while the XCL model performed best with an AUC of 0.8585 and sensitivity of 0.8125. In the GHN external test group, the AUC of the XCL model was 0.8553 (Fig. 5, Table 3).

**Table 3:** Comparison of NRI and IDI between ML model (XGBoost, CatBoost, LightGBM, and XCL model) and EuroSCORE II.

	NRI			IDI		
	Value	95%CI	*P*	Value	95%CI	*P*
Internal test group						
XGBoost - EuroSCORE II	0.8177	0.3564–1.2791	<0.001	0.1084	0.0250–0.1918	0.0108
CatBoost - EuroSCORE II	1.0100	0.5719–1.4481	<0.001	0.1151	0.0362–0.1940	0.0042
LightGBM - EuroSCORE II	0.9776	0.5603–1.3950	<0.001	0.1359	0.0550–0.2186	<0.001
XCL model - EuroSCORE II	1.2339	0.9380–1.5298	<0.001	0.1473	0.0628–0.2318	<0.001
External test group of QLH						
XGBoost - EuroSCORE II	0.5552	0.2097–0.9007	<0.001	0.0330	0.0001–0.0658	0.0490
CatBoost - EuroSCORE II	0.6868	0.3486–1.0250	<0.001	0.0724	0.0165–0.1282	0.0115
LightGBM - EuroSCORE II	0.6024	0.2548–0.9500	<0.001	0.0326	0.0007–0.0655	0.0500
XCL model - EuroSCORE II	0.7669	0.4351–1.0988	<0.001	0.0729	0.0374–0.1044	<0.001
External test group of GHN						
XGBoost - EuroSCORE II	0.3857	−0.1876–0.0959	0.1872	0.0114	−0.0262–0.0035	0.1329
CatBoost - EuroSCORE II	0.7678	0.1929–1.3426	0.0088	0.0204	−0.0195–0.0602	0.3116
LightGBM - EuroSCORE II	0.2273	−0.3475–0.8021	0.4383	0.0228	−0.0187–0.0644	0.2818
XCL model - EuroSCORE II	0.4685	0.1255–1.0625	<0.001	0.0293	−0.0184–0.0570	0.3156

Abbreviation: NRI, net reclassification improvement; IDI, integrated discrimination improvement; EuroSCORE, European System for Cardiac Operative Risk Evaluation; CI, confidence interval.

Additionally, the optimal threshold for dividing patients into low- and high-risk mortality groups was determined by using the cutoff point where both sensitivity and specificity were maximized, based on the maximum Youden index (Table 3).

### 3.3 Calibration

The Hosmer-Lemeshow (H-L) goodness-of-fit test shown that all models demonstrate good to excellent calibration (*P* > 0.05) in the internal test group. However, in the external test groups, particularly with QLH and GHN, some models show significant calibration issues (especially XGBoost and LightGBM), while others like the XCL model maintain strong calibration. The XCL model generally shows good to excellent calibration across all groups, indicating its robustness and reliability (Table 4).

**Table 4:** Hosmer-Lemeshow goodness-of-fit statistic.

	Model	*P* value
Internal test group		
XGBoost		0.181
CatBoost		0.261
LightGBM		0.185
XCL		0.296
EuroSCORE II		0.359
External test group of QLH		
XGBoost		0.021
CatBoost		＜0.001
LightGBM		0.092
XCL		0.548
EuroSCORE II		0.172
External test group of GHN		
XGBoost		＜0.001
CatBoost		0.069
LightGBM		＜0.001
XCL		0.244
EuroSCORE II		0.043

Abbreviation: QLH, Qilu Hospital of Shandong University; GHN, General Hospital of Ningxia Medical University.

**Fig. 6:**
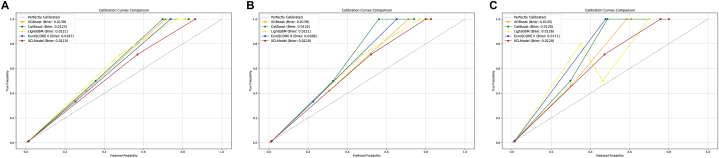
The calibration plots of the risk evaluation models in (A) the internal test group, (B) the external test group of QLH and (C) the external test group of GHN.

The calibration plots provide a more visual indication of the calibration of the model. The calibration of the five models is acceptable both in the internal test group and in the external test group. However, all five models underestimated the mortality of high-risk patients (Fig. 6).

### 3.4 Consistency analysis

**Fig. 7:**
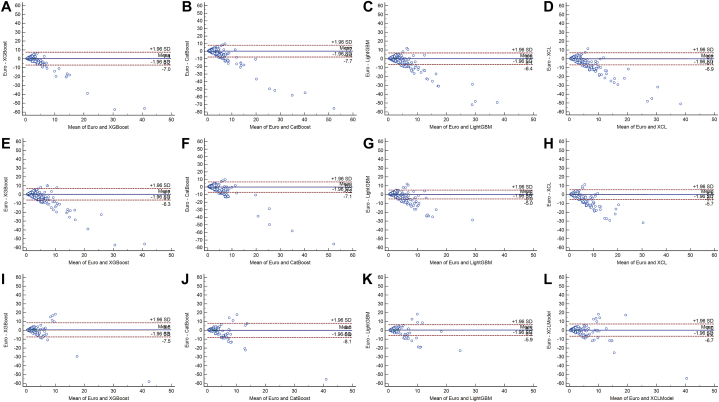
The Bland-Altman plots for in-hospital mortality prediction. (A–D) Consistency tests between machine learning models and the EuroSCORE II in the internal test group; (A) The consistency test between XGBoost and the EuroSCORE II; (B) The consistency test between CatBoost and the EuroSCORE II; (C) The consistency test between LightGBM and the EuroSCORE II; (D) The consistency test between XCL-Modle and the EuroSCORE II. (E–H) Consistency tests between machine learning models and the EuroSCORE II in the external test group of QLH; (E) The consistency test between XGBoost and the EuroSCORE II; (F) The consistency test between CatBoost and the EuroSCORE II; (G) The consistency test between LightGBM and the EuroSCORE II; (H) The consistency test between XCL-Modle and the EuroSCORE II. (I–L) Consistency tests between machine learning models and the EuroSCORE II in the external test group of GHN; (I) The consistency test between XGBoost and the EuroSCORE II; (J) The consistency test between CatBoost and the EuroSCORE II; (K) The consistency test between LightGBM and the EuroSCORE II; (L) The consistency test between XCL-Modle and the EuroSCORE II.

The agreement between the four ML models and EuroSCORE II was good in the internal test set (Fig. 7).

The consistency between the four ML models and EuroSCORE II performed relatively well in the Shandong and Ningxia external test groups (Fig. 7).

### 3.5 Clinical benefits

All four ML models performed better than EuroSCORE II within a threshold probability of 0–5 %.

**Fig. 8:**
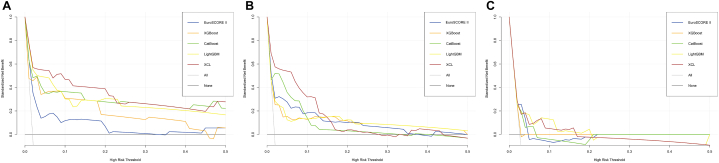
Decision curve analysis (DCA) of the risk evaluation models plotting the net benefit at different threshold probabilities. (A) DCA of the models in the internal test group; (B) DCA of the models in the external test group of QLH; (C) DCA of the models in the external test group of GHN.

In the internal test group, the four ML models show a definite advantage over EuroSCORE II, regardless of the threshold probability of the segment. There was no significant difference in performance between the four ML models. In the two external test groups, the advantage was not significant (Fig. 8).

### 3.6 NRI and IDI

NRI and IDI are two new evaluation metrics to assess the degree of improvement of the model over the another. If the values of NRI and IDI are positive, then it suggests that the model improves over the other. Conversely, it implies a negative improvement. The ML models showed positive gains in all three test groups compared to EuroSCORE II (Table 2).

### 3.7 Explanation of XCL model with the SHAP method

To identify the importance of risk factors, SHapley Additive exPlanations **(**SHAP) values were calculated. SHAP link game theory to local explanations and interprets the output of any ML model in a unified way. Fig. 2 shows the weights of risk-factors importance. The former (Fig. 2A) showed the importance of risk factors for predicting mortality after CABG operation. In order of importance, they were preoperative estimated glomerular filtration rate (eGFR), preoperative left ventricular ejection fraction (LVEF), intra-aortic balloon pump (IABP) implantation, body mass index (BMI), age, surgical status, bypass graft number, CAD classification, cardiopulmonary bypass, New York heart association (NYHA) IV, diabetes, morbid obesity, gender, cerebrovascular disease, pulmonary hypertension, previous percutaneous coronary intervention (PCI), number of diseased vessels, peripheral vascular disease, hypertension, valvular disease, chronic obstructive pulmonary disease (COPD), and atrial fibrillation respectively. The latter (Fig. 2B) showed the importance of the value of each variable in predicting mortality after the CABG operation. In the figure, red dots indicate higher values of the variables, which are associated with an increased risk of mortality, while blue dots represent lower values, which are associated with a reduced risk. For example, for “Preoperative eGFR,” lower values (represented by blue dots) tend to push the SHAP value to the left (negative impact), indicating a higher mortality risk when eGFR is low. Fig. 3 shows the individual plots for patients who did not survive and survived. The SHAP values indicated risk factors and their contribution to the prediction of patient mortality. Where f(x) was the predicted value of mortality, red indicated risk factors that increased mortality, and blue indicated risk factors that decreased mortality, where longer arrows indicated a greater degree of impact on mortality.

## 4 Discussion

The ability to predict in-hospital mortality is acceptable for both traditional risk evaluation model EuroSCORE II and ML models. All four ML models demonstrate advantages over EuroSCORE II in internal and external validation. In particular, the XCL model based on the Bagging strategy performed best and was stable.

XGBoost, CatBoost, and LightGBM are three classical ML methods [18,21,22], and the three base learners are combined in an ensemble learning manner to form the XCL model. Theoretically, the ensemble nature of the XCL model, inherently allows it to capture complex relationships and interactions between variables. This capability likely contributed to its robust performance, even when tested on populations with varying characteristics. All four ML models outperformed the traditional EuroSCORE II in terms of discrimination, calibration, and clinical benefit. The XCL model showed a definite advantage, significantly beating the other three base learners.

ML models may better minimize the training error but often tend to over-fit the data [28]. It can make ML models perform too perfectly when validated internally. One of the essential aspects of ML models to predict the risk of cardiac surgery is the generalization of the prediction efficiency of models. Theoretically, the modeling and test groups are the same sample, and the excellent performance of the risk evaluation model in the test group is predictable and understandable. However, the model's practicability should be reliably verified in broader, real-world settings [29].

Data from two different regions were used for external validation to address this concern. China has a vast territory, and the medical level and disease spectrum of patients in each region differ. A distance of more than 500 km can be considered a different area. The distance between the two externally verified hospitals is more than 11000 km. The observed differences in patient characteristics (e.g., age, comorbidities) are expected in real-world clinical settings. The purpose of external validation is precisely to test the model's ability to generalize across different populations with varying baseline characteristics. These differences between the modeling group and the external test groups are reflective of natural patient variability and demonstrate the model's robustness in handling diverse populations. Despite the differences in characteristics between the groups, the XCL model maintained strong predictive performance in the external test groups, as demonstrated by its consistent AUC, calibration, and decision curve analysis results. This indicates that the model was able to effectively generalize across different patient populations, suggesting that the variability in characteristics did not significantly impact its predictive ability.

Machine learning models like XCL hold promise for transforming clinical decision-making by allowing for personalized, data-driven risk assessments. Expanding the scope of the XCL model to predict not only in-hospital mortality but also long-term outcomes and postoperative complications would further enhance its utility [15,30]. For example, clinicians could use such models to identify patients at risk of specific complications like atrial fibrillation or kidney injury [31], enabling early interventions and tailored care plans.

Incorporating these models into clinical workflows requires the development of user-friendly tools that can provide clinicians with easily interpretable predictions [32]. This could involve the use of threshold scores, as shown in this study, to identify high-risk patients and guide perioperative decision-making. By integrating ML models with electronic health records (EHRs) and clinical support systems, clinicians could access real-time predictions to optimize treatment strategies and improve patient outcomes.

In recent years, more and more researchers have tried to explain ML models by using the feature attribution framework of SHAP [33]. With SHAP to explain the XCL model, several variables associated with in-hospital mortality after CABG surgery were identified. In this study, the preoperative eGFR was recognized as the most important predictor variable. Lower eGFR was associated with increased mortality risk. Reduced kidney function is a well-established risk factor for adverse outcomes after cardiac surgery [34], as patients with renal impairment are more prone to complications, including infections and heart failure.

Other critical variables identified through SHAP analysis included preoperative LVEF, IABP implantation, BMI, and age. Lower preoperative LVEF was associated with higher mortality, highlighting the vulnerability of patients with reduced cardiac function [35]. These patients may have more difficulty recovering from the physiological stress of CABG surgery, increasing the risk of postoperative complications. IABP implantation was another significant predictor, often used for high-risk patients needing mechanical circulatory support [36]. Its association with increased mortality risk is not surprising, given that these patients typically present with severe cardiovascular compromise. Higher BMI values were also associated with increased mortality, consistent with existing research linking obesity to higher rates of postoperative complications, such as infection and wound healing problems [37]. Finally, age emerged as a key predictor, with older patients being more susceptible to poor outcomes due to reduced physiological reserves and the presence of multiple comorbidities.

## 5 Limitations

First, the retrospective observational analysis leads to bias due to its inherent drawbacks. Second, the lower mortality rate and fewer positive events during patient hospitalization in the training set can make the ML model less capable of predicting the occurrence of positive events. Third, the time of this study is large and early cases would have been excluded from the study due to missing data. Fourth, this study's focus on an Asian cohort restricts the generalizability of findings to other ethnic groups.

## 6 Conclusions

The ML models have significant advantages over EuroSCORE II in predicting postoperative in-hospital mortality in CABG patients. In particular, the XCL model showed potential benefits in the internal and external test groups. This novel analytical approach may provide reliable information on cardiac operation in the near future.

## Ethics approval

This study was reviewed and approved by The Ethics Committees of Jiangsu Province Hospital, The Ethics Committees of Shanghai East Hospital, The Ethics Committees of Shanghai Chest Hospital, The Ethics Committees of Qilu Hospital of Shandong University, and The Ethics Committees of General Hospital of Ningxia Medical University with the approval number: 2022-SR-464, IS22063, 2017018, KYLL-202208-030, and KYLL20220584, dated 2022-08-30, 2022-08-17, 2017-06-12, 2020-10-01, 2022-09-15.

## Availability of data and materials

We are pleased to share data. The data involved in our research are available from the corresponding author. We will respond in 7 days on reasonable request.

## Funding information

This research received no grant from any funding agency in the public, commercial, or not-for-profit sectors.

## CRediT authorship contribution statement

**Kai Xu:** Writing – review & editing, Writing – original draft, Visualization, Methodology. **Lingtong Shan:** Writing – original draft, Validation, Software. **Yun Bai:** Writing – original draft, Software, Formal analysis, Data curation. **Yu Shi:** Validation, Software, Methodology. **Mengwei Lv:** Methodology, Investigation, Data curation. **Wei Li:** Supervision, Software, Resources. **Huangdong Dai:** Validation, Supervision, Software. **Xiaobin Zhang:** Visualization, Validation. **Zhenhua Wang:** Validation, Supervision, Software. **Zhi Li:** Validation, Supervision, Software. **Mingliang Li:** Writing – review & editing, Writing – original draft, Supervision. **Xin Zhao:** Writing – review & editing, Writing – original draft, Project administration, Methodology, Investigation, Data curation, Conceptualization. **Yangyang Zhang:** Writing – original draft, Validation, Supervision, Project administration, Conceptualization.

## Declaration of competing interest

The authors declare that they have no known competing financial interests or personal relationships that could have appeared to influence the work reported in this paper.
